# Children and war: the work of the Children and War Foundation

**DOI:** 10.3402/ejpt.v4i0.18424

**Published:** 2013-01-15

**Authors:** William Yule, Atle Dyregrov, Magne Raundalen, Patrick Smith

**Affiliations:** 1 Department of Psychology, Institute of Psychiatry, King's College London, London, UK; 2 Center for Crisis Psychology, Fortunen, Bergen, Norway

**Keywords:** Children, war, disasters, evidence-based interventions

## Abstract

The Children and War Foundation was established after the authors’ experiences following the civil war in former Yugoslavia in the mid-1990s. Many organizations tried to mitigate the effects of the war on children but few interventions were based on evidence and fewer were properly evaluated. The Foundation was established in Norway with the aim of promoting better evidence-based interventions to help children after wars and natural disasters.

The Foundation has developed a number of empirically grounded manuals that aim to help children learn strategies that will lessen the stress reactions that they have developed. The manuals are designed to be delivered by personnel who are not necessarily very experienced in child mental health. They are aimed at groups of children using a public health approach to reach large numbers in a short space of time. The strategies are not intended as individual therapy.

The *Teaching Recovery Techniques* manual has been used following a number of earthquakes and other natural disasters and data from a number of these will be discussed. A *Writing for Recovery* manual is aimed at helping adolescents and is based on the seminal work of James Pennebaker. It is currently being evaluated in three separate studies. A group-based manual to help children bereaved by war or disaster has recently been developed.

It is difficult to reconstruct the reactions and feelings of children exposed to the conflict in World War II (Stargardt, 2010). The contemporary data base consists of very patchy written records and their representativeness remains unknown. During this period, child mental health professionals had very different conceptual frameworks compared to today. In the 1940s, they were still heavily influenced by psychoanalytic constructs, which have failed to stand the test of time. Moreover, Stargardt pointed out that even Anna Freud was highly selective in using records to support her theories rather than examining the evidence in an unbiased fashion.

From contemporary official records, it is clear that in the 1940s, both lay and professional, people were totally divided on to how best to help children deal with their reactions to war experiences. How should they advise children to deal with distressing intrusive memories? Should they encourage children to talk about them, or to suppress them? There were no empirical findings to guide them.

Things had not improved greatly by the time of the outbreak of the civil war in Yugoslavia 50 years later. There were very few studies on how to ameliorate the psychological distress of children. However, there was one big difference: the United Nations had been created and among its relevant agencies, UNICEF, WHO and UNHCR were stimulating professionals to develop appropriate ways of responding. Indeed, in 1993, UNICEF appointed a Psychosocial Advisor (a Norwegian clinical psychologist called Rune Stuvland) to coordinate the psychosocial projects there.

A number of child mental health professionals were recruited by UNICEF to alert politicians and educators of the nature of the harm that war would inflict on children. These included Atle Dyregrov, William Yule, Robert Pynoos, Anica Kos and others who had experience of working with traumatized children. In a meeting in Otocec in Croatia in July 1993, the group shared their impressions of what was happening. Many children were being affected but there were no screening procedures to identify those most in need of help—for the simple reason that few appropriate screening methods were available. Even more alarming, while there were many well-intentioned people volunteering to help, none had any evidence that what they were proposing to do would do more good than harm. Put simply, intervention projects were rarely properly evaluated. The group decided that there was a need to develop simple screening measures as well as evidence-based interventions.

It was these decisions that resulted in our decision to form the Children and War Foundation. It was intended to be a Foundation that would attract large amounts of money so that it could fund social and clinical scientists to develop better ways of screening children's needs; to develop evidence-based group interventions; and of evaluating outcomes.

## The Convention on the Rights of Children

A major development since the 1940s has been the development of the Convention on the Rights of the Child (CRC) (United Nations [UN], 1989). Given that modern warfare deliberately targets women and children and that the numbers who are displaced as a result of war have soared exponentially, it is worth noting a few of the key Articles that relate to the welfare of war affected children.

Key Articles in the CRC include:

- Article 9—the right not to be separated from the parents
- Article 24—right of access to health care
- Article 28—right to education
- Article 39 states—parties shall take all appropriate measures to promote physical and psychological recovery and social reintegration of a child victim of any form of neglect, exploitation or abuse: torture or any other form of cruel, inhuman or degrading treatment or punishment; or armed conflict. Such recovery and reintegration shall take place in an environment which fosters the health, self-respect and dignity of the child.

Thus, signatories to the convention were entreated to respect some basic rights of children including providing appropriate interventions to promote their psychological recovery.

The 1996 report of the UN Secretary General on the Impact of Armed Conflict on Children (The Machel Report) firmly concluded that psychological recovery and social reintegration must be a central feature of all humanitarian assistance programs. The report stated that programs aimed at relieving psychological suffering must take into account the social and cultural context of children and their families.

Such documents, however well received initially, are often shelved. Graca Machel revisited the situation and produced an updated report in 2009 (UNICEF, 2009). In the intervening period in the 1990s, more than 2 million children had died in armed conflicts. Twenty million children were at that time displaced. From the point of view of a child, it matters little whether they have been “internally displaced” or have crossed an international boundary and might claim asylum. They just experience multiple losses. In addition, over 300,000 children had been recruited as child soldiers. The world-wide situation had become more dire.

However, the follow-up Machel report (UNICEF, 2009) reflected another change. By then, many charities and NGOs had begun to offer help to war affected children. Unfortunately, a rift appeared between many of these organizations and the rapidly developing evidence base for interventions. Whereas in the 1940s there had been considerable conceptual problems around the concept of “trauma”—was it an intra-psychic phenomenon or an environmental assault—in the late 1990s, many NGOs regarded any discussion of “trauma” in children as reflecting a pathologizing and medicalizing of human experience. Some saw the offer of individual treatments as imposing Western constructs on developing nations. It was seen as an extension of Western imperialism and colonization. While cultural issues should always be addressed, something undefined called “traditional healing” was lauded (Inter-Agency Standing Committee [IASC], 2007).

It is true that it was only after the Vietnam War that people began to make sense of the drastic reactions that many battlefield survivors developed. “Lack of moral fiber” in the face of the enemy, shell shock and so on came to be understood as common reactions to life threatening experiences. The concept of post-traumatic stress disorder was codified in 1980 to capture the main elements (American Psychiatric Association [APA], 1980). At first it was assumed that children did not develop post-traumatic stress disorders (PTSD) but that was unfounded because the data base did not include direct interviews with children. Adults denied that children would be affected by major trauma or if they were, they were so resilient that it could be ignored. By the mid-1990s, we had substantial evidence that children exposed to war and disaster did indeed develop a number of post traumatic reactions which ranged much wider than PTSD. However, the core reactions within PTSD were a useful way of summarizing complex reactions.

While the debate raged (usually with little recourse to evidence, IASC, 2007), very effective and efficient methods of intervention were being developed for adults. Our Foundation cooperated with others to ensure that children's needs were not overlooked.

## The situation in 2012

Currently, it is accepted that some children do develop PTSD after experiencing very stressful, life-threatening events such as happen in war. Warfare in the 21st century is very different from even that experienced during World War II. There are many more guerrilla-type civil wars in which women and children are not only the main victims, but are deliberately targeted. Thousands are displaced both internally and across borders. Thus, any programs that intend to mitigate the psychological effects of such trauma need to adopt a public health approach aimed at reaching many thousands. However sophisticated and effective individual therapies may be, they need to be adapted to deliver to many survivors.

Individual therapies for PTSD in adults have developed significantly since the 1980s. The UK National Institute for Clinical Excellence (2005) reviewed the evidence from clinical trials and was able to recommend both Trauma-Focused Cognitive Behavior Therapy (TF–CBT) and Eye Movement Desensitization and Reprocessing (EMDR) therapy for adults. While less well supported (because of far fewer studies) only TF–CBT was recommended for children. NICE found little evidence to support the use of drugs in treatment, and none to support generic “counseling” or art or music therapies. These are still promoted by many NGOs after a disaster or war.

However, the evidence base for any form of group intervention is small. In war, there is no one single traumatic event and children may be exposed to many war related events over many months or even years. Displacement from their homes, separation from their families and disruption to schooling, all affect children's mental health. Hence, group interventions have to be adapted to take these factors into consideration.

With individual TF–CBT being endorsed to help adults who have developed PTSD (Ehlers, Clark, Hackmann, McManus, & Fennell, 2005), it has also been adapted to form the basis of interventions with children who have been exposed to peacetime single event traumas (Smith, Perrin, Yule, & Clark, 2010; Smith et al. 2007). All clinicians need to be sensitive to cultural considerations. The available evidence suggests that traumatic stress reactions are more similar across cultures than they are different (e.g., Zhang, Zhang, Wu, Zhu, & Dyregrov, 2011). What is needed is better international agreement on how best to respond to mental health needs following complex emergencies (Mollica et al., 2004).

One of the constant dilemmas is when to intervene. After a civilian, one-off traumatic event, how quickly should psychological help be offered? Received wisdom suggests that early intervention should be better than delayed intervention. However, there is now some evidence that if the intervention is too early, it may disrupt natural healing processes in adults (e.g., Dyregrov & Regel, 2012; Mayou, Ehlers, & Hobbs, 2000) and appears ineffective in preventing persistent PTSD symptoms when used with children (e.g., Stallard et al., 2006; Zehnder, Meuli, & Landolt, 2010). In contrast to single session early interventions for all trauma-exposed children, a screen-and-intervene approach in which a more intense multi-session early intervention is provided for symptomatic children appears promising (Berkowitz, Stover, & Marans, 2011).

In a war or post-war situation, the acute stage will usually have long passed and so there is less reason to delay offering help once it is safe to do so. Should that help be offered to everyone, or should it be targeted at those most badly affected? If it is to be targeted, then one needs reliable, valid and sensitive screening methods to identify those at greatest risk.

Where there are many hundreds, indeed thousands, of affected survivors, it will usually prove impossible to provide one-to-one therapy, even if that has been shown to be effective. In most war situations, there is likely to be a great shortage of trained child mental health practitioners. Therefore, ways have to be found to deliver services by people less well qualified. Fortunately, in most countries there are determined efforts to get children back in to schools and so it makes sense to consider delivering appropriate child mental health support through schools and even teachers.

Thus, a fully comprehensive approach to delivering mental health services after war should take into consideration how best to support children, their families and schools through community based initiatives. Some teachers may be given additional training in mental health techniques and within school, group intervention sessions can be arranged. In this step-wise approach, successive steps act as screens to identify children requiring more specialized interventions and so only a few very seriously affected children would be offered individual work.

Given that wars will be with us for the foreseeable future, we need to learn, and learn quickly, to provide more effective and efficient help for children. As Mollica et al. (2004) pointed out, there is a great need to undertake research in this area. At the very least, all interventions should be evaluated to learn what works best and also to stop wasting resources on interventions that either do no good or even do harm.

## Developing measures

In January 1994, four of us (Atle Dyregrov, Rune Stuvland, Bob Pynoos, and Bill Yule) met in Bergen to take the UNICEF projects forward. We agreed to develop measures that would screen for adverse stress reactions in children and that would be sensitive to change so that they could also be used pre- and post-interventions and so evaluate them. With no specific funding, we adapted existing measures and obtained permission from copyright holders to do so. We considered that the reactions of children to stress are broad and so we worked on measures of anxiety, depression and traumatic stress. We are indebted to Mardi Horowitz and Peter Birleson for permission to adapt their pioneering measures of stress reactions (the Impact of Event Scale) and depression, respectively.

Bill Yule was commissioned by UNICEF to help re-establish child mental health services in Mostar in the South of Bosnia-Herzegovina. Together with Patrick Smith and later with Sean Perrin, David Schwartz and Berima Hacam, school personnel were helped in devising a series of training seminars for teachers to help them address the changes in children's behavior after the conflict. A few teachers were given supervision to provide mental health interventions and 15 years later they are still doing so.

Patrick Smith undertook a survey of the mental health needs of 3,000 children in Mostar using the early series of measures. Not surprisingly, it was documented that the children had experienced many traumas, with over 50% reporting they and been shot at, 50% saying they had seen dead bodies, and 25% saying they had witnessed a killing. They reported high levels of post-traumatic stress on the Impact of Event Scale (Smith, Perrin, Yule, & Rabe-Hesketh, 2001). The series of measures was later used in a study of Bosnian refugees in Macedonia, which again documented high levels of depression and stress (Papageorgiou et al., 2000).

## The Children and War Foundation

We had been shocked at how little evidence was available to guide people in selecting effective interventions to help children after wars. It was puzzling given the enormous resources expended by governments and NGOs to alleviate stress. We came to realize that there were systemic reasons for this unacceptable situation. When a crisis erupts, there is a great deal of publicity. Graphic scenes are shown on television and appeals are made for money. It is too late by then to prepare for the crisis (as Mollica et al., 2004 recommend)—it is upon us and everyone tries to do his or her best. As the crisis recedes (or falls off the television screen), the publicity spotlight moves away and the money goes with it. In other words, there are no resources to develop better ways of working or to evaluate what was done.

This was the gap that the Children and War Foundation was formed in 2001 to fill. It was established in Norway with funds donated by Atle Dyregrov, Magne Raundalen and Bill Yule. Harald Kobbe, an attorney at law with long-established links with UNICEF joined us and the fifth Board member was the Norwegian Children's Ombudsman, Trond Waage. It took a long time to gather sufficient funds to be able to sponsor high quality studies. For the most part, as described below, we have focused on three areas: (1) the development of measures; (2) the development of manualized interventions; and (3) support for relevant small studies.

## Measures

From the beginning, the Foundation realized that it could not directly manage intervention projects. We also realized that there was a great need for training suitable people to deliver help on a large scale. Since most wars and disasters occur in resource poor areas, we further determined to give away our products as cheaply as possible. Thus, with the agreement of the original copyright holders, we have made measures that are available to download free from our website. We have encouraged people to undertake proper translations and back translations and to share their findings with us. The translated measures are then added to the website. At present, the Children's Revised Impact of Event Scale exists in around 20 different languages.

We are aware that many people wishing to help children may be unfamiliar with the need to obtain valid measures. They do not want long, unwieldy questionnaires. To that end, we undertook factor analytic studies of large data sets and produced an 8-item short version, which has been shown to have good reliability and validity and to be sensitive to change (Perrin, Meiser-Stedman, & Smith, 2005).

Gradually we have added other measures and made them available. Another gap in measures available to professionals was a measure of complicated grief reactions. These often occur when a child witnesses the sudden death of a loved one during conflict. Our new Traumatic Grief Inventory for Children was shown to have good psychometric properties in a study in Belgium (Dillen, Fontaine, & Verhofstadt-Deneve, 2009). It was successfully used in the intervention study in Iran (Kalantari et al., 2012). Thus, we have met one of our aims—to develop and disseminate better ways of assessing children's needs.

## Interventions

In May 1998, six people who had been working in former Yugoslavia and Africa were brought together by the Foundation and met for 3 days on an island near Bergen. We brainstormed what was known about effective, evidence-based methods to boost children's capacity to cope with the psychological aftermath of war. The aim was to produce a manual that could be used with minimal training and supervision by people responsible for the well-being of children affected by war and disasters. The intervention was to be delivered in groups so that large numbers of children could be helped in a short time. The aim was not to “cure” symptoms but rather to give children better coping strategies so that they would feel sufficiently more in control of their reactions and so be able to benefit from the support of their families and the opportunities available in schools.

The deliberations were written up in manual form. This is the *Teaching Recovery Techniques* manual (Smith et al. 2007) that can be obtained on request via our website. We had determined that we would not release the manual until positive field trials had been achieved, but under pressure, we allowed colleagues in Greece and Turkey to use it following the big earthquake of 1999. Hence, we developed two versions of the manual—one for wars and one for disasters. The results were very positive (e.g., Giannopoulo, Dikaiakou, & Yule, 2006). We strongly advise that people using the manual should have some training in it beforehand. We have trained a number of senior trainers in London and Bergen who can do this, and so far have trained more than 300 others in its use in various countries such as Malaysia, Thailand (for SE Asia), Sri Lanka, Kenya, China, Bangladesh, Georgia and Japan as well as Norway and the UK. The manual gets revised on the basis of feedback from users.

A second manual, *Writing for Recovery*, (Yule et al. 2005) was developed in a similar way. For many years, Jamie Pennebaker (cf. Pennebaker, 1993) in Austin, Texas has shown how powerful the use of writing about an emotional experience can have beneficial health effects. Arnold van Emmerik in the Netherlands had shown that a structured writing program helped adults with PTSD, and Frank Neuner in Germany had shown that Narrative Exposure Therapy was helpful in dealing with the traumatic reactions to multiple stressors among groups of refugees in Uganda and children in Sri Lanka. Together with Atle Dyregrov, Bill Yule and Magne Raundalen, they developed a structured writing manual that can be used with groups of adolescents. The adolescents meet twice a day for three consecutive days. Each meeting lasts for only 15 min (a total of only 90 min of writing). They are asked to write about their innermost feelings about an index traumatic event. The instructions change subtly to direct them first to their psychological reactions and latterly to what they have learned that was most helpful. A pilot study of the effects on Iraqi adolescent refugees in Jordan showed that it had at least short term effects and this is now the subject of a large randomized controlled study (Ali & Snell, in progress).

The manual was also used in Iran with bereaved Afghani adolescent refugees. In a randomized control study (Kalantari, Yule, Dyregrov, Neshatdoost, & Ahmadi, 2012), those who received the writing intervention showed far less distress than the waiting list control condition on the Foundation's Traumatic Grief Inventory for Children. However, findings are mixed. The randomized controlled trial of Lange-Nielsen et al. (2012) found no effect of the writing intervention on the self-reported PTSD symptoms of adolescents in Gaza.

Recently, we have developed a third group intervention to help children with bereavement reactions following loss as a result of war or disasters (Dyregrov, Yule, Straume, & Kraus, 2012). Given the enormous variation in mourning rituals and beliefs, the first draft of the manual was evaluated by professionals from many different cultures, with very positive endorsements. We will now sponsor field trials and, assuming success, we will make it available for use.

## Empirical studies

As noted earlier, the Foundation helps develop the infrastructure for others to mount evidence-based interventions. We have supported an increasing number of studies that will not only provide help to children but will also yield much needed data on which to base decisions regarding the efficacy of different interventions. Colleagues seeking how best to intervene regularly consult us and we encourage them to work closely with UNICEF and other local government structures. We encourage them to train key individuals who can then cascade the training to others and so reach a large number of children. We emphasize the need to get measures before and after intervention to evaluate the effects. Some have been able to mount controlled and randomized controlled trials. The manuals have also been used following large scale natural disasters. Gudarzi and Yasamy (2007) cascaded training to hundreds of teachers, psychiatrists and psychologists so as to provide help to over 21,000 survivors of the devastating earthquake in Bam, Iran in 2003.

The *Teaching Recovery Techniques* manual was first used in 1999 following the devastating earthquakes in Turkey and Greece. Working in a child mental health service in Athens, Giannopoulo et al. (2006) demonstrated that the techniques were easy to use with small groups of children and that the measures showed significant improvement in adjustment. Unfortunately, the Turkish data have not yet been published in peer reviewed journals.

The same manual was used with asylum seeking adolescents in London (Ehntholt, Smith, & Yule, 2005). It was found that participation in the groups was initially helpful but the effects soon dissipated. These adolescents were also experiencing the current stress of awaiting decisions on their asylum applications. Further trials are currently underway.

Following training organized by UNICEF in Malaysia after the Tsunami, the Child Trauma Psychosocial Response Team led by Dr Lai Fong Hwa adapted the manual for use with children in Penang which had been badly hit by the 2004 Asian Tsunami. Similarly, after training in London, Dr Charles Sim was posted to Myanmar after Cyclone Nargis in 2008. Together with Maria Plengsantip, he conducted and evaluated a group for 26 children aged 6–17 years. They were able to mount a 6-month follow-up and reported positive findings to a meeting of the International Society for Traumatic Stress Studies.

A number of Georgian psychologists received training in the use of the Teaching Recovery Techniques at the Centre for Crisis Psychology in Bergen in December 2008. They used their new skills to help children affected by the civil war in Georgia and published their positive experiences in a symposium in Tbilisi in October 2010 (Gedevanishvili, 2010).

At the request of the Sri Lankan Education Department and the collaboration of GTZ (the German government department for overseas aid), Bill Yule trained 120 teacher counselors working in the North and East of Sri Lanka. This was after the Tsunami and still during the civil war. The teachers reported a very high level of stress reactions and bereavement in the children, but without the benefit of using any measures. One of the challenges was that the traumatic events, often including sudden displacement, were still on-going. Despite the difficulties, these dedicated teachers worked with many thousands of children. Unfortunately, the planned formal evaluation had to be abandoned because of the fighting and political situation. Such reality factors indicate why it has been so difficult to evaluate interventions in the real world.

Although it is obvious to most of us that children are adversely affected by witnessing wars and violence, it still proves necessary to demonstrate to authorities and donors just how serious the problems can be. This is where our standardized measures can help. Working through Oasis Africa, Dr Gladys Mwiti was funded to study the effects of the 2007 post-election violence in Kenya. A total of 882 adolescents aged 12–19 years participated and 63% were found to have scores above the cut-off points that indicated a high risk of having serious stress reactions. Subsequently, the Foundation held a training event for approximately 50 participants in Nairobi and the *Teaching Recovery Techniques* manual is currently being used there.

After participating in a training event held by us in Bangkok, Dr Nuttorn (submitted) undertook a randomized controlled study of the use of the manual with children who had been affected by the 2004 Asian Tsunami. Even a few years after the traumatic event, the manual had a significant effect in reducing stress reactions. That study is currently awaiting publication.

The Foundation funded CARE Palestine to implement a randomized control trial as well as to roll out the training to many teachers (Barron, Abdullah, & Smith, 2012). In a school-based RCT, adolescents (*N*=133) from the West Bank in Palestine were randomly allocated to participate immediately in TRT groups run by local counselors, or put on a waiting list. Significant reductions in PTSD symptoms (*d*=0.76), depression (*d*=1.24), and grief (*d*=0.96) were found in the intervention group relative to the waiting list).

Quota, Palosaari, Diab, and Punamaki (2012) conducted a large (*N*=482, 10–13 years old) school-based RCT of the TRT manual compared to regular school lessons. Results were mixed. The intervention significantly reduced the proportion of clinical PTSD symptoms among boys, and among girls who had low levels of dissociation. The authors discuss the implications of these findings for delivering risk-specific interventions for a range of children traumatized by war.

From the results of small case studies, small controlled trials and larger randomized control trials, we are building up good evidence for the efficacy of this approach. It does not reduce all psychological problems. We are moving to a situation where we can deconstruct the package and examine which elements are the most powerful. It may be that eventually we can produce a streamlined version that will allow many more war-affected children to be helped quickly.

This wish to provide ever more efficient ways of delivering helps lay behind our decision to develop the *Writing for Recovery* manual. A small controlled study in Pakistan following an earthquake showed that even just three writing sessions had a positive effect in reducing stress and improving memory (Kamal, 2008). As noted earlier, the writing intervention was also very effective in reducing bereavement reactions among war-affected adolescents.

The manuals have been used in a number of other masters and doctoral dissertation studies. In two cases where the manual was adapted rather than used in its entirety, the effects were not as good. In another case, circumstances determined that the investigators were unable to be present in the conflict zone and relied on others to implement the intervention. Again, the results were disappointing. Such experiences suggest that there are active elements in the package that may be identified by deconstruction studies, but that for the time being, adherence to the manual is strongly recommended.

The Foundation has also funded a number of studies of basic processes underlying stress reactions. These are now reaching publication stage (Neshat Doost, Yule, Kalantari, Rezvani, Dyregrov, & Jobson, submitted; Neshat Doost, Dalgleish, Yule, Kalantari, Ahmadi, Dyregrov, & Jobson, 2013).

## The future

Like all charities, our continuing activities depend on raising funds and that is increasingly difficult in the current economic climate. Having established ourselves and now getting many enquiries for advice and help, we may have to rethink our earlier decision not to manage intervention projects directly. It is easier to get donations when one can show pictures of children being helped—far less easy if one just shows some questionnaires and manuals.

Despite this, we feel that our decision to develop better ways of assessing children's needs and of evaluating interventions was the right one. The development of intervention manuals has been exciting and is now paying off. It would be marvelous to think that our measures and manuals would not be needed to help war affected children in the foreseeable future but history shows that for many years to come, children will still be the victims of armed conflicts.
